# Hypertrichose nævoïde

**DOI:** 10.11604/pamj.2013.15.56.2941

**Published:** 2013-06-20

**Authors:** Naoufal Hjira, Mohammed Boui

**Affiliations:** 1 Service de dermatologie, hôpital militaire d'instruction Mohammed V, Rabat, Maroc

**Keywords:** Hypertrichose nævoïde, hypertrichose congénitale, cheveux, lasers épilatoires, nevoid hypertrichosis, congenital hypertrichosis, hair, laser hair removal

## Image en médicine

L'hypertrichose nævoïde est une forme rare d'hypertrichose congénitale localisée, elle se manifeste par une croissance excessive de poils terminaux. Les lésions cutanées sont présents dès la naissance ou apparaissent juste après, le plus souvent elles sont solitaires, des cas de lésions multiples étaient également rapportés. La texture et la couleur des poils est celle des cheveux du cuir chevelu, rarement ils sont gris ou blancs. Elle est le plus souvent isolée, toutefois elle peut être associée à des atteintes systémiques : oculaires, neurologiques, musculaires et une dysmorphie faciale, d'où l'intérêt d'explorer tout patient présentant une hypertrichose nævoïde à la recherche d'anomalies systémiques associées. Les lésions peuvent persister à vie, cependant des régressions spontanées étaient rapportées. Le traitement fait appel aux les lasers épilatoires. Nous rapportons le cas du nourrisson de sexe féminin âgée de 9 mois, qui nous a été adressé pour évaluation de deux lésions cutanées au niveau du cuir chevelu évoluant depuis la naissance. L'examen clinique trouvait un nourrisson sans dysmorphie. L'examen cutané objectivait deux touffes de cheveux sur les régions temporales émergeant d'un cuir chevelu de couleur normale. Nous avons cru au début que c'est la coiffure rituelle de certaines tribus marocaines. Mais l'interrogatoire révélait que la fille n'a jamais subi de coiffure. Le reste de l'examen physique n'a pas objectivé de lésions extra-cutanées. L'examen histologique montrait un épiderme normal, avec un nombre élevé de follicules pileux morphologiquement normaux dans le derme. Le diagnostic était celui d'une hypertrichose nævoïde. L'attitude thérapeutique était l'abstention et la surveillance.

**Figure 1:**
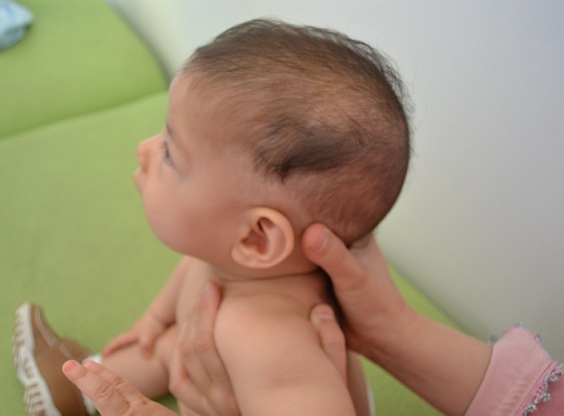
Cheveux en touffe à croissance excessive sur la région temporale

